# Characterization of the complete mitochondrial genome of *Neoasterolepisma foreli* (Insecta: Zygentoma: Lepismatidae) and the phylogeny of basal Ectognatha

**DOI:** 10.1080/23802359.2020.1848480

**Published:** 2021-01-13

**Authors:** Claudio Cucini, Antonio Carapelli, Claudia Brunetti, Rafael Molero-Baltanás, Miquel Gaju-Ricart, Francesco Nardi

**Affiliations:** a Department of Life Sciences, University of Siena, Siena, Italy; b Department of Zoology, University of Cordoba, Cordoba, Spain

**Keywords:** Silverfish, Zygentoma, mitogenomics, myrmecophily, Lepismatidae

## Abstract

The silverfish *Neoasterolepisma foreli* belongs to the family Lepismatidae within Zygentoma and is well known for the peculiar habit of living in strict association with ant nests (myrmecophily). In this study, we describe its mitochondrial genome, a circular molecule of 15,398 bp including the canonical 13 PCGs, 22 tRNAs, 2 rRNAs, as well as a 403 bp AT-rich region. A phylomitogenomic analysis of the new sequence, alongside basal hexapod mtDNAs, confirmed the monophyly of all orders, with some uncertainty over the position of the enigmatic *Tricholepidion gertschi* that would make Zygentoma paraphyletic. *Neoasterolepisma foreli* is recovered in a basal position within family Lepismatidae, at odd with our current understanding of the group that would, in turn, suggest a closer relationship with the genus *Lepisma* (Mendes, 1991).

Zygentoma (silverfishes) is a small taxon of primitively wingless hexapods. Despite the availability of anatomical and embryological data (Gaju-Ricart et al. 2015), genetic and genomic information is still limited. They are generally regarded as sister group of the flying insects (e.g. Mendes 2018), with some doubts on the enigmatic *Tricholepidion gertschi* that may represent an early offshoot of the common dicondylian stock, making Zygentoma the only non-monophyletic high-ranking basal hexapod group. This latter hypothesis was proposed based on morphological evidence (i.e. Boudreaux 1979; but see Kristensen 1998; Blanke et al. 2014) and revived based on phylomitogenomic investigations (Carapelli et al. 2006; Leo et al. 2019). Lepismatidae are the largest family within Zygentoma, display a cosmopolitan distribution and include well known anthropophilic species such as *Lepisma saccharinum* and *Thermobia domestica.* Nevertheless, infraorder relationships are still unclear. The species *Neoasterolepisma foreli* (Moniez, 1894) has been investigated in relation to its myrmecophilous habit (Molero-Baltanás et al. 2017), but its phylogenetic position has never been studied in detail. In this work, we describe the complete mitochondrial genome of *Neoasterolepisma foreli* and study its phylogenetic position within basal Dicondylia.

A single specimen of *N. foreli* collected in 2019 in Vélez de Benaudalla (Granada, Spain; 36°50'13″N 3°30′24″W; voucher ID: NFO_03, preserved at the Life Sciences Department of the University of Siena; determined by M. G. -R.) was used for total genomic extraction using QIAmp^®^ UCP DNA kit. Libraries were prepared using the TruSeq DNA Nano kit (Illumina, San Diego, CA) and 151 bp paired-end sequences were obtained on a HISeq 2500 platform (Illumina, San Diego, CA) at Macrogen Europe together with additional libraries from unrelated arthropod species (data not shown). Resulting reads were *de novo* assembled as in Nardi et al. (2020). In brief, the *cox3* gene was employed as seed in the NOVOPlasty v. 3.8.3 (Dierckxsens et al. 2017) assembling method with manually curated *k* = 33 and 39. The resulting assemblies were checked against a MEGAHIT assembly (Li et al. 2015) and manually curated. The final mtDNA sequence was automatically annotated using MITOS (Bernt et al. 2013) and manually curated. The new sequence (PCGs only) was retro-aligned with the dataset of Leo et al. (2019), together with *Ctenolepisma villosum* (Chen et al. 2019) and *Lepisma saccharinum* (Bai et al. 2020) sequences, and hypervariable regions were removed using Gblocks v. 0.91b (Castresana 2000). The best partitioning scheme and evolutionary model were selected using PartitionFinder 2.1.1 (Lanfear et al. 2016) and used for a Bayesian phylogenetic analysis in MrBayes 3.2.6 (Ronquist et al. 2012) through the CIPRES Science Gateway (Miller et al. 2010) with four chains of 2 × 10^7^ generations, a burn-in of 0.25 and a sampling frequency of one tree every 1000 iterations.

The complete mtDNA of *N. foreli* is 15,398 bp long, in line with the previously described mitogenomes of Lepismatidae (e.g. *Thermobia domestica*: 15,152 bp, *Lepisma saccharinum*: 15,244 bp, *Ctenolepisma villosum*: 15,488 bp). The molecule encodes for the common 37 genes encountered in metazoan mtDNAs (13 PCGs, 22 tRNAs, and 2 rRNA) plus a non-coding A + T rich region of 403 bp. Overall, all PCGs show a canonical Methionine start codon (ATN), with the exception of *cox1* (TTG – Leucine), *nad3* and *nad5* (ATT – Isoleucine). All PCGs are characterized by a TAA complete stop codon, with the exception of *nad1* (TAG), *nad3* and *cob* (T–). Two overlaps between adjoining genes are observed, namely *atp8–atp6* (4 nt) and *nad4*–*nad4L* (7 nt). Gene order conforms to the ancestral Pancrustacea model (Boore 1998), as in all other silverfishes studied (Comandi et al. 2009). The nucleotide composition of the entire mitogenome is as follows: A = 39.8%, T = 32.2%, C = 17.6% and G = 10.4%, with the highest AT nucleotide bias (72%) within the order (Cook et al. 2005; Comandi et al. 2009; Chen et al. 2019; Bai et al. 2020).

The phylogenetic tree obtained from the Bayesian Inference analysis confirms the results of Leo et al. (2019) and, despite the addition of new sequences, most of the inter-order relationships remain unvaried. A comparison with Chen et al. (2019) and Bai et al. (2020) is, on the other hand, more difficult due to a different taxon sampling in these latter. Overall, the tree supports the monophyly of Ectognatha, Dicondylia, Pterygota, Ephemeroptera, Odonata, Microcoryphia and Diplura with high posterior probability values, but not of Zygentoma (Figure 1). In fact, as shown in previous morphological and molecular studies (Leo et al. 2019; Montagna 2020), *T. gertschi* appears to occupy a basal position within the Dicondylia clade, hence making Zygentoma paraphyletic with respect to Pterygota, although with marginal support. If any, this indicates that the phylogenetic position of this relict species is still a debatable topic (Blanke et al. 2014). At the family level, Machilidae (Microcoryphia) is recovered as paraphyletic with respect to Meinertellidae due to the position of *Petrobiellus puerensis* (nomen nudum) and *P. bannaensis* (nomen nudum) as sister group to *Nesomachilis australica*. Within bona fide Zygenoma (i.e. Euzygentoma), *Atelura formicaria*, the only representative of family Nicoletiidae family, is sister group to the Lepismatidae. *Neoasterolepsima foreli* appears as the basal group within Lepismatidae at odds with our current understanding of the group that would suggest a closer relationship with *Lepisma* (Mendes, 1991). The study of new samples and the review of previously published ones would be warranted to clarify the position of *N. foreli* and *L. saccharinum.*

**Figure 1.**
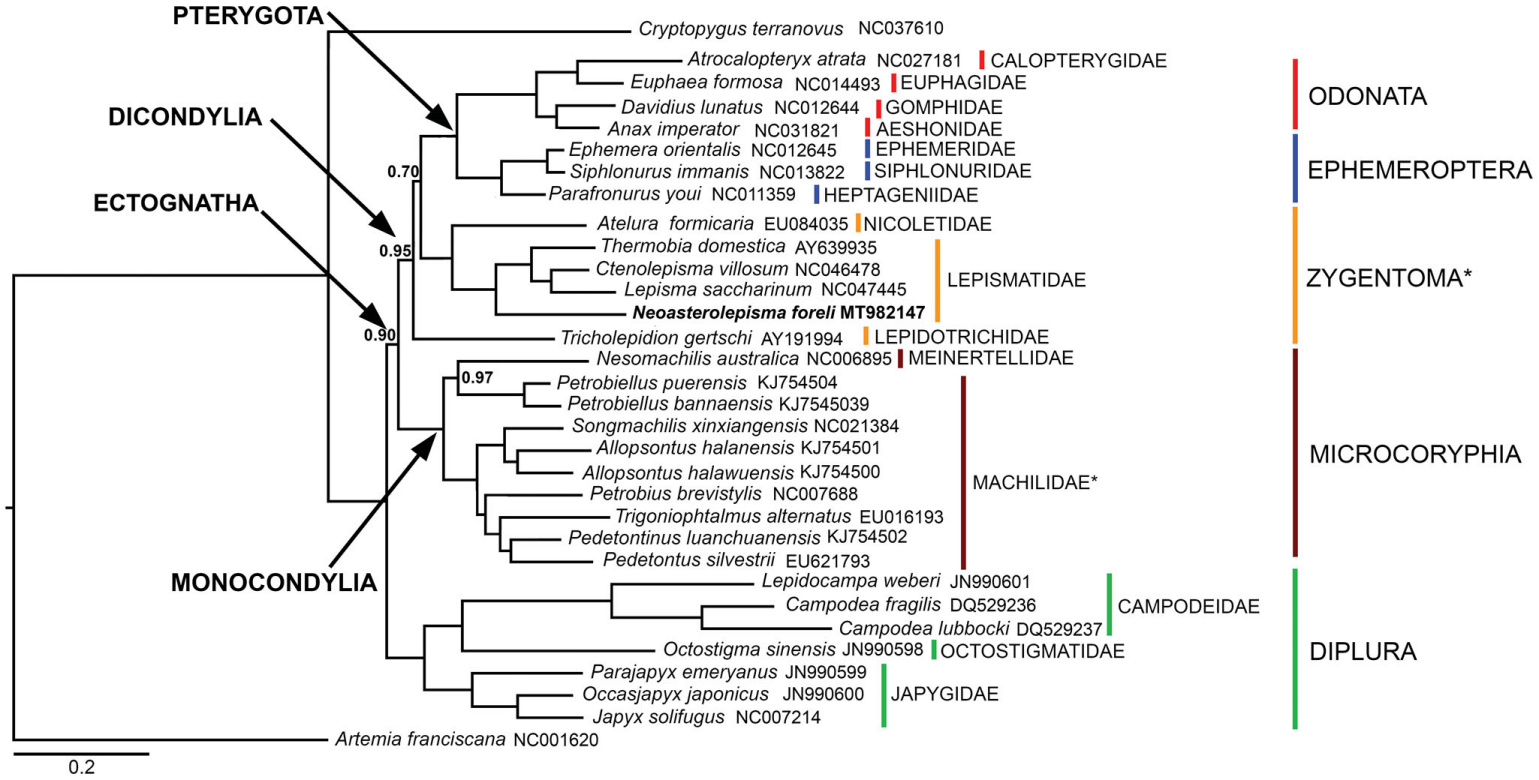
Phylogenetic tree obtained through a Bayesian statistical approach on the concatenated 13 PCGs of basal Ectognatha and two outgroups (*Artemia franciscana* and *Cryptopygus terranovus*). *Neoasterolepisma foreli* phylogenetic position is bold highlighted. Paraphyletic groups are highlighted with an asterisk. Posterior probabilities are shown at nodes (full support if not otherwise indicated).

## Data Availability

Mitogenome data supporting this study are openly available in GenBank at: https://www.ncbi.nlm.nih.gov/nuccore/MT982147. Associated BioProject, SRA, and BioSample accession numbers are https://www.ncbi.nlm.nih.gov/bioproject/PRJNA673074 https://trace.ncbi.nlm.nih.gov/Traces/sra/?study=SRP289641 https://www.ncbi.nlm.nih.gov/biosample/SAMN16587686.
